# Brazilian consensus recommendations on the diagnosis and treatment of light chain amyloidosis

**DOI:** 10.1016/j.htct.2026.106482

**Published:** 2026-06-02

**Authors:** Pedro Manoel Marques Garibaldi, João Tadeu Damian Souto Filho, Roberto Jose Pessoa de Magalhães Filho, Roberta Shcolnik Szor, Carlos Eduardo Miguel, Edvan Crusoe, Carolina Tosin Bueno, Thiago Queiroz, Vânia Hungria

**Affiliations:** a Division of Hematology, Department of Medical Imaging, Hematology, and Clinical Oncology, Ribeirão Preto Medical School, University of São Paulo, Ribeirão Preto, Brazil; b Faculdade de Medicina de Campos, Campos dos Goytacazes, Rio de Janeiro, Brazil; c Instituto Oncomed de Educação e Pesquisa, Rua Lopes Trovão, 318, Niterói, Rio de Janeiro, Brazil; d Rede Americas, Hospital 9 de Julho, São Paulo, Brazil; e Foundation of the Regional Medical School of São José do Rio Preto (FAMERP), São José do Rio Preto, Brazil; f Instituto D'or Oncologia, Brazil and Federal University of Bahia, Salvador, Brazil; g Johnson & Johnson Innovative Medicine, São Paulo, Brazil; h Department of Hematology and Oncology, Clínica São Germano, São Paulo, Brazil

**Keywords:** Amyloidosis, Light chain, Brazil, Consensus, Recommendations

## Abstract

**Introduction:**

Systemic immunoglobulin light chain amyloidosis is characterized by the extracellular deposition of misfolded light chains, leading to progressive dysfunction of multiple organs. Diagnosis remains a significant clinical challenge and is frequently delayed due to the presence of nonspecific symptoms, coupled with limited access to specialized diagnostic tools and expertise. Currently, no established national recommendations for the diagnosis and management of this condition exist in Brazil.

**Methods:**

These expert recommendations were developed following a structured advisory meeting in May 2025. The guidelines were formulated after a national survey of 96 Brazilian institutions (50 private and 46 public). Key diagnostic and therapeutic challenges were identified, including delayed diagnosis, limited access to biopsy techniques and biomarker testing, and uneven availability of treatment options. Recommendations focused on areas where consensus would benefit healthcare professionals in establishing best practices.

**Results:**

The recommendations included: 1) Early detection: Educational initiatives for patients and healthcare professionals and the establishment of multidisciplinary specialized centers are needed to improve time to diagnosis and subsequent prognosis. 2) Accurate diagnosis: Clear, standardized procedures for tissue biopsy and timing are necessary to ensure accurate diagnosis and efficient testing. Improved access to Congo red staining and polarized light microscopy is critical. 3) Evaluation and classification: Biomarker testing should be used at all stages. Validated biomarker cut-offs and scoring systems should be implemented to standardize patient evaluation and risk stratification. 4) Optimal management and treatment: A multidisciplinary approach is crucial. Daratumumab should be added, if available, to bortezomib, cyclophosphamide and dexamethasone. Bortezomib and melphalan based regimens are an alternative.

**Conclusion:**

These expert-guided recommendations aim to support earlier diagnosis, standardized evaluation, and optimized treatment of light chain amyloidosis in Brazil.

## Introduction

Amyloidosis is a heterogeneous disorder of protein misfolding and metabolism in which insoluble fibrils are deposited in various tissues (Supplementary Table 1), causing organ dysfunction and eventually death [1,2]. Immunoglobulin light chain amyloidosis (AL) is characterized by the excessive production of abnormal and unstable antibody light chains by a proliferative plasma cell or B cell clone. In the systemic disease, light chains produced by clonal cells in the bone marrow accumulate in multiple organs, particularly in the heart and kidneys [1,3,4]. Systemic AL amyloidosis is progressive and has high morbidity and mortality rates, particularly when diagnosis is delayed and significant organ damage has occurred [5,6]. Diagnosing and treating AL amyloidosis early is therefore critical to reduce or eliminate the light chain-producing plasma cell clone and halt organ damage [4,7, 8, 9].

### Epidemiology and patient journey to diagnosis

AL amyloidosis is a relatively rare condition, with a worldwide incidence of 5.1–12.8 cases per million population [1,7]. Systemic disease is more common, with localized disease representing just 7–12% of all AL amyloidosis cases [5]. Median age at diagnosis is 64 years with incidence increasing with age; however there is no established association with ethnicity or geography [1]. Pre-existing plasma cell dyscrasias, including MGUS and multiple myeloma, carry a significant risk of progression to AL amyloidosis [10,11]. In 2025, a new definition of light chain MGUS was proposed, with the aim of decreasing the overdiagnosis of monoclonal gammopathies often linked to using the standard reference intervals for serum free light chains (sFLCs) [12]. With the new definition of light chain MGUS based on these revised, more accurate reference intervals, the false-positive rate of free light chain (FLC) testing decreased by 82% [12]. New reference intervals for sFLCs in individuals with chronic kidney disease have been created from the large prospective population-based cohort of the iStopMM study. Using the current reference intervals, 60% and 21% of patients had abnormal kappa and lambda sFLC values, respectively. Novel reference intervals (based on central 99% reference intervals) have been proposed for serum kappa FLC (mg/L), serum lambda FLC (mg/L), and the sFLC ratio in individuals with estimated glomerular filtration rates (eGFR) of 45–59, 30–44, and < 30 mL/min/1.73 m². However, it should be noted that these new ranges were established in a homogeneous cohort; therefore, they may not be fully generalizable to more diverse or wider populations [13]. These updates to the definition of MGUS will directly impact the diagnostic workflow (detection of the monoclonal component) and, in turn, the need for biopsies to detect amyloid deposits [14].

Light chain infiltration leads to multiorgan damage and dysfunction [7]. There are two forms of organ involvement: deposition of insoluble protein leading to organ dysfunction (primarily in the heart and kidneys) and direct tissue toxicity from circulating soluble-free light chains (e.g. elevated N-terminal pro-brain natriuretic peptide [NT-proBNP] values indicative of cardiac stress dissociated from structural alterations) [15]. Symptoms appear after organ damage has already occurred and are related to the organs involved; most patients have multi-organ involvement at the time of diagnosis [7,16,17]. One-year mortality is high (12%–30%), reflecting the failure to diagnose prior to irreversible end organ damage [9,18]. Heart involvement is the major determinant of prognosis and mortality [4,7]. Cardiac dysfunction results from proteotoxicity of the light chains and amyloid deposits that cause widespread disruption of tissue architecture [19].

### Current treatment landscape

Advances in treatments of AL amyloidosis in the last 40 years have increased the median overall survival from 1.4 years in the 1980s to 4.6 years in the 2010s, with a growing number of patients achieving survival of ten years or longer [9,20,21]. Improvements in survival have lagged in patients with cardiac involvement. Deaths result from disease-related factors with cardiac failure (32%) and sudden unexpected death (23%) being the leading causes [9]. Overall survival in the 2010s was 8.8 years for patients with no cardiac involvement compared with 2.6 years in patients with cardiac involvement [9]. With improved long-term survival (29% of deaths occurring > 10 years after diagnosis), non-AL amyloidosis-related death has increased from 3% to 16% of the total deaths [9]. However, most patients who survived at least ten years required one or more subsequent lines of therapy [20], and patients continued to experience significant and persistent symptom burden after diagnosis, particularly while on active treatment [22].

## Developing practical guidance for diagnosing light chain amyloidosis in Brazil

The incidence of AL amyloidosis in Brazil is estimated to be 6.72 cases per million population [23], though data on AL amyloidosis are scarce. However, physicians are increasingly aware of the disease because of the growing number of publications, mainly from American, European, and Asian centers [24]. In Brazil, amyloidosis referral centers have emerged in recent years, with an aim to improve the diagnosis and treatment of patients [24,25]. Several guidelines and consensus recommendations exist for the diagnosis and management of AL amyloidosis worldwide, including regional versions, e.g., for the UK and Singapore [4,26, 27, 28, 29, 30]. However, there are no Brazilian guidelines or recommendations.

## Objective

The purpose of this article is to provide expert-guided practical guidance and recommendations for the optimal diagnosis, evaluation and classification, and treatment of AL amyloidosis in Brazil. This article aims to educate and aid specialists and community physicians in Brazil who are involved in the treatment of patients with AL amyloidosis, as well as wider clinical teams who may support the early diagnosis of patients (including specialists such as cardiologists, nephrologists and neurologists).

## Methods

These expert recommendations were formulated following a virtual structured advisory exchange on May 21, 2025. The objective of the meeting was to discuss the latest and most significant data for AL amyloidosis. Advisors in Brazil were selected based on their significant expertise in hematology and extensive experience managing AL amyloidosis within both academic and real-world settings. Selection criteria also included a distinguished record of peer-reviewed publications and active participation in AL amyloidosis clinical trials.

During the meeting, leading experts shared practical, real-world insights regarding the management of AL amyloidosis in Brazil, specifically focusing on: diagnosis (clinical manifestations and investigations); evaluation and classification (techniques, staging, and prognosis); and therapeutic strategies (treatment principles, the current landscape, and response evaluation). The topics selected were areas where consensus recommendations would be highly beneficial to healthcare professionals (HCPs) managing patients with AL amyloidosis and where alignment is required to establish best practices. In particular, the consensus topics and recommendations were informed by the results of the survey conducted at 96 institutions across Brazil (North, Northeast, Central-West, Southeast, and South) to identify the diagnostic and therapeutic challenges in AL amyloidosis; the survey questions are summarized in Table 1.

**Table 1:** Questions from a survey conducted at 96 institutions across Brazil to identify the diagnostic and therapeutic challenges in light chain amyloidosis.

Survey Title: Diagnosis and Treatment of light chain Amyloidosis (AL) in Brazil – Mapping Challenges and Possible Pathways	
Question	Possible responses
1) How many cases of AL amyloidosis are diagnosed annually at your institution?	0–5
	6–10
	11–20
2) In your clinical practice, what is the average time between the onset of the first signs/symptoms and the diagnosis of AL amyloidosis?	< 6 months
	7–12 months
	13–24 months
	> 24 months
3) Do you have easy access to different specialties (such as cardiology, nephrology, neurology) for multidisciplinary diagnostic and therapeutic management of patients with AL amyloidosis?	Yes
	No
4) In your clinical practice for the multidisciplinary diagnostic and therapeutic management of patients with AL amyloidosis, which specialties do you have easy access to?	Cardiologist
	Nephrologist
	Neurologist
	Dermatologist
	Gastroenterologist
	Gastrointestinal surgeon
	Hepatologist
	Interventional radiologist
	Quaternary hospital
5) Which of the following tests for detecting the monoclonal components are available in your clinical practice for investigating AL amyloidosis?	Serum protein electrophoresis
	Serum immunofixation
	Urinary immunofixation
	dFLC
	None of the above
6) Which of the following tissues are available for collection in your clinical practice for the purpose of identifying amyloid material?	Bone marrow
	Abdominal fat
	Tongue
	Myocardium
	Kidney
	Peripheral nerve
	Gastrointestinal tract
	Salivary gland
	Liver
7) Which technique is used for abdominal fat collection?	Incisional biopsy
	Punch biopsy
	Core needle biopsy
	Fine needle aspiration of abdominal fat
	None of the above
8) Which of the following techniques are available in your clinical practice for anatomopathological analysis and identification of amyloid material?	Congo red staining
	Polarization with polarized light (birefringence)
	None of the above
9) Which of the following amyloid protein typing techniques are available in your clinical practice?	Immunohistochemistry
	Immunofluorescence
	Immunoelectron microscopy
	Mass spectrometry
	None of the above
10) Is mass spectrometry performed at your institution or are the samples sent to a reference center?	Performed internally
	Sent to national reference laboratory
	Sent to international center
	I do not know
11) Which of the following tests are available in your clinical practice for prognostic and organ dysfunction assessment in patients with AL amyloidosis?	NT-proBNP
	Troponin (T or I)
	Echocardiogram with GLS
	Echocardiogram without GLS
	Cardiac MRI
	Scintigraphy
	24-hour proteinuria
	None of the above
12) Which of the following therapies are available in your clinical practice for the treatment of patients with AL amyloidosis?	Autologous HSCT
	Melphalan
	Cyclophosphamide
	Proteasome inhibitors (e.g., bortezomib, carfilzomib)
	Anti-CD38 antibody (e.g., daratumumab)
	BCL-2 inhibitors (e.g., venetoclax)
	Thalidomide
	Lenalidomide
	None of the above (referral to relevant service, we do not perform any chemotherapy)

AL: immunoglobin light chain amyloid protein; BCL-2: B-cell lymphoma 2; CD: cluster of differentiation; dFLC: difference between involved and uninvolved free light chains; GLS: global longitudinal strain; HSCT: hematopoietic stem cell transplantation; MRI: magnetic resonance imaging; NT-proBNP: N-terminal pro-brain natriuretic peptide.

Expert consensus and recommendations were obtained using elements of the Nominal Group Technique (NGT), which allows simplicity, structured discussion, and immediate consensus [31]. To implement the NGT, the meeting was structured to first review key data around each topic. This review was followed by a discussion in a round robin format to allow sequential view sharing and equal contribution from all advisors. Group discussion occurred at the end of the round robin to allow time for questions, clarification, and consolidation of key recommendations.

Recommendations generated are based on the available clinical evidence, as well as clinical experience and knowledge gained through daily practice of the participating experts in the field of AL amyloidosis. Although the best-practice recommendations are intended to be a flexible tool to assist timely and informed decisions, they should not replace sound clinical judgment or be used as a legal resource.

## Light chain amyloidosis diagnosis

### Difficulties in early detection

AL amyloidosis is a heterogeneous disease with the potential to affect multiple organs, resulting in broad and variable clinical presentations that complicates diagnostic characterization [32]. The diagnosis is delayed as symptoms typically emerge only after organ damage has accumulated to the point of dysfunction. Increased serum FLC levels may be present for at least four years before the onset of clinical manifestations [7,33]. Early symptoms of AL amyloidosis are vague (i.e., neurologic symptoms, fatigue, dizziness, abdominal pain) and may be misattributed by patients and HCPs to other, more common conditions or simply as part of aging [16,34,35]. Once symptoms become more serious, their heterogenous nature may further contribute to misdiagnosis and diagnosis delay [36]. In a retrospective study of patient medical records, patients had a median of five precursor diagnoses within the two years prior to diagnosis [35]. The median time from symptom onset to AL amyloidosis diagnosis is approximately six months to 2.7 years with 50% of patients visiting five or more physicians of different medical specialties before diagnosis [16]. HCPs involved in diagnosis include, but are not limited to, primary care providers, medical internists, cardiologists, nephrologists, neurologists, gastroenterologists and hematologists [34]. Notably, patients with renal involvement often experience a more direct diagnostic pathway and are frequently diagnosed within six months of symptom onset, likely reflecting the routine use of kidney biopsy in patients with proteinuria. In contrast, delayed diagnosis is particularly consequential in patients with cardiac involvement, for whom early recognition is critical to improving outcomes [32].

The clinical presentation of AL amyloidosis depends on organ involvement, most commonly the heart, kidneys, nervous system, soft tissues, and liver [37] (Supplementary Figure 1, Supplementary Table 2). Symptoms more specific to AL amyloidosis are not common and present later in the disease, further complicating diagnosis [4,16,34]. Symptoms that should immediately raise suspicion of possible AL amyloidosis include the involvement of soft tissues with macroglossia, periorbital purpura, submandibular gland swelling, and the shoulder pad sign; however, these highly specific symptoms are rare and occur only in a subset of individuals [37,38]. In the absence of more specific symptoms, physicians should suspect AL amyloidosis when patients present with heart failure with preserved ejection fraction, nephrotic-range proteinuria in the absence of diabetes, peripheral neuropathy, unexplained hepatomegaly, and diarrhea, especially when multiple organ systems are involved [38] (Supplementary Figure 1, Supplementary Table 2).

### Diagnostic testing

**Figure 1:**
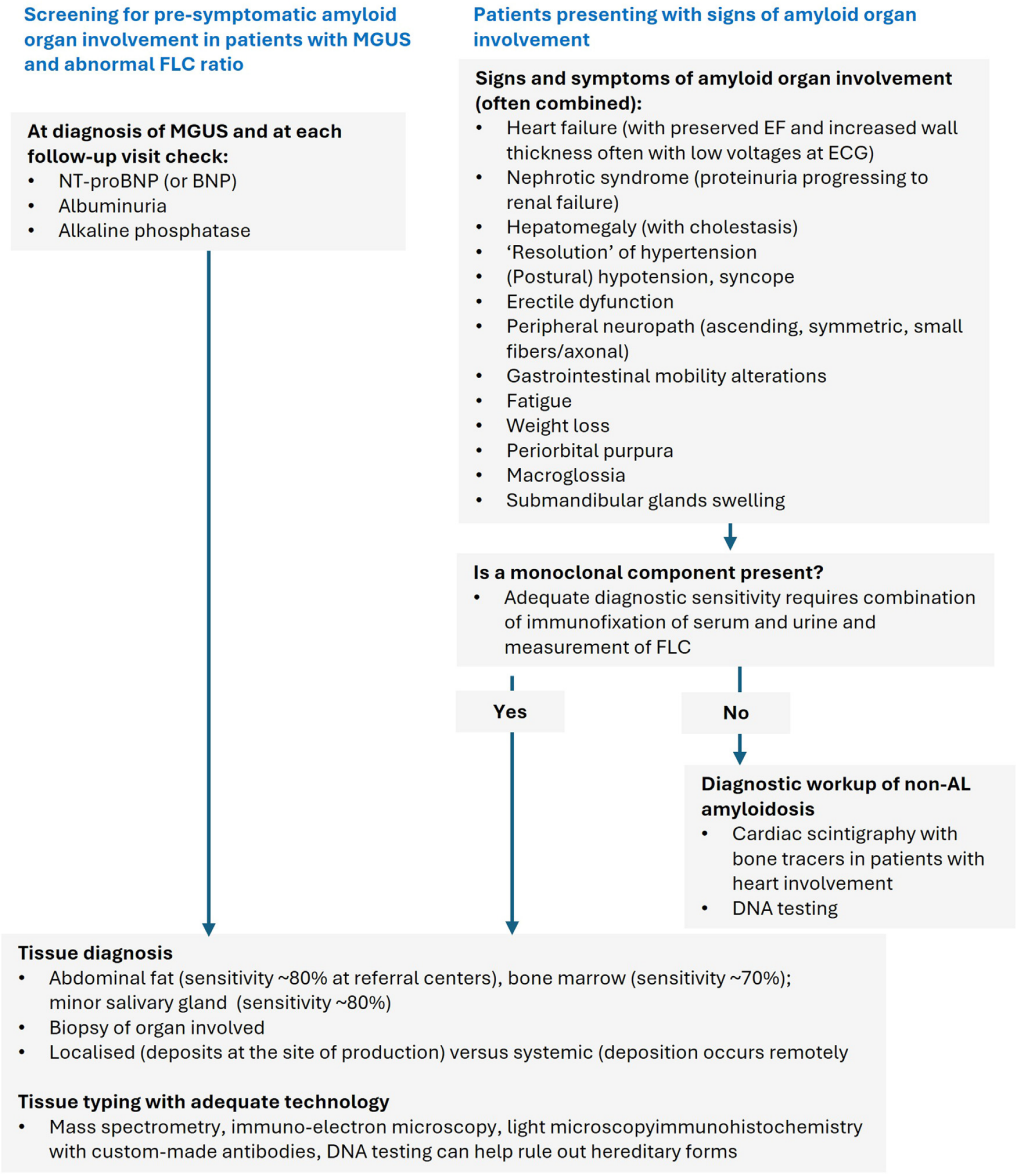
Diagnostic algorithm for AL amyloidosis Adapted from Palladini [4] AL: Immunoglobin light chain amyloid protein; BNP: B-type natriuretic peptide; ECG: electrocardiogram; EF: Ejection fraction; FLC: Free light chain; MGUS: Monoclonal gammopathy of undetermined significance; NT-proBNP: N-terminal pro-B-type natriuretic peptide.

Assessment for AL amyloidosis is indicated when there is a clinical suspicion of the disease due to the presence of common symptom patterns (Supplementary Figure 1, Supplementary Table 2). Patients with pre-existing conditions that increase the risk of acquiring AL amyloidosis would benefit from regular screening for biomarkers of the disease that may increase before symptoms appear. Biomarkers, including alkaline phosphatase (ALP), proteinuria, and NT-proBNP, should be monitored regularly in patients with MGUS to detect early signs of organ involvement [39]. Patients are assessed for AL amyloidosis using a 4-step algorithm: 1) Screening for clinical suspicion of AL amyloidosis, 2) identification of monoclonal light chains, 3) tissue diagnosis, and 4) typing of tissue employing adequate technologies (Figure 1) [7,8,40]. The presence of plasma cell dyscrasia in patients with suspected AL amyloidosis is determined by the identification of monoclonal light chains by three tests: serum protein immunofixation, urinary immunofixation, and the serum kappa/lambda ratio [4,8]. Because the clonal plasma cell population in AL amyloidosis is often small ( < 10% of total bone marrow cells), protein electrophoresis is frequently negative and therefore usually omitted from the diagnostic algorithm [4,8]. Once a monoclonal component has been detected, the presence of AL amyloidosis requires the identification of amyloid fibrils in a tissue biopsy. This confirmation is based on the demonstration of amorphous tissue material that stains positive with Congo red and exhibits the characteristic apple-green birefringence under polarized light, a critical step to avoid both false-positive and false-negative interpretations in the routine practice [4,41,42]. Congo red is a relatively inexpensive dye with a long shelf-life that rebinds all forms of amyloid [41]. The initial evaluation typically relies on combined sampling of peripheral tissues, such as abdominal fat aspirate or biopsy, bone marrow, or salivary gland, which are more accessible and carry lower procedural risk. However, negative fat and bone marrow biopsies do not rule out AL amyloidosis; there is still an approximate 15% chance of disease and hence further biopsies of the appropriate organs are recommended [18]. Biopsies of clinically affected organs provide higher sensitivity and specificity but are less readily available and associated with a higher risk of complications, such as perioperative bleeding of which kidney biopsies pose a significant risk [4,18,43]. The sensitivity of peripheral tissues in detecting amyloid deposits can be lower in the clinical practice than that demonstrated by the literature (e.g., abdominal fat pad biopsy). In individuals who are highly suspected of having AL amyloidosis and have an absence of amyloid deposits in peripheral tissues, biopsy of an affected organ should be prioritized. According to the American Society of Hematology (ASH) guidelines on the diagnosis of AL amyloidosis, the combination of fat pad sampling and bone marrow biopsy yields a high sensitivity (89%). When performed individually, however, sensitivity is significantly lower (76.6% and 55.1%, respectively), whereas endomyocardial biopsy demonstrates a sensitivity of 93.5%. However, these guidelines recognize that proceeding with one approach over the other is dependent on several external factors including resource access, available expertise, clinical presentation, and associated costs [13]. Once amyloid deposits have been confirmed with Congo red testing, the type of amyloid protein must be identified in a tissue biopsy using more specific methods such as immunological techniques or mass spectrometry. Genetic testing may also contribute to subtyping the amyloidosis in hereditary cases [44]. The choice of the subtyping technique depends on ease of referrals, funding availability, cost, and access to specialized resources, including specific antibodies and mass spectrometry expertise [44].

In the absence of a monoclonal component, patients with suspected cardiac amyloidosis should be tested for transthyretin (ATTR) amyloidosis using cardiac scintigraphy with bone-seeking tracers, notably ^99m^Tc-diphosphono-propanodicarboxylic acid, ^99m^Tc-pyrophosphate, and ^99m^Tc-hydroxymethylene diphosphonate [4,45]. A DNA analysis is necessary to differentiate between hereditary and wild-type ATTR amyloidosis and to dismiss other rarer hereditary forms [4,45].

### Risk stratification

Patients diagnosed with AL amyloidosis undergo risk stratification and disease staging based on the type and extent of organ involvement to help determine prognosis and guide treatment decisions. Over recent decades, improved diagnostic accuracy and therapeutic advances have led to a better understanding of the natural history of AL amyloidosis and contributed to better overall outcomes [9]. Nevertheless, prognosis remains strongly influenced by the diagnostic timing and the presence and severity of cardiac involvement – a key determinant of both treatment feasibility and survival. Cardiac complications, particularly progressive heart failure and malignant arrhythmias, remain the leading causes of morbidity and mortality in AL amyloidosis [9]. The current staging systems therefore rely heavily on measures of cardiac function and circulating biomarkers of cardiac damage [8,29]. Cardiac biomarkers assessed in these models include levels of troponin T (TnT) and NT-proBNP or brain natriuretic peptide (BNP) [46]. Additional criteria used for disease staging include an assessment of clonal disease based on the difference between involved and uninvolved circulating FLC levels (dFLC); however, in the daratumumab era, this may not be as prognostically important [18]. Cardiac biomarkers alone might be more suitable to stage AL amyloidosis as baseline FLC showed no effect on the depth of response in newly diagnosed patients [47]. A renal staging system, in which renal involvement is assessed as the eGFR level and degree of proteinuria, helps predict the risk of progression to dialysis [46].

Staging models that utilize soluble cardiac biomarkers include the Mayo 2004 model, the European 2015 modification of Mayo 2004 model, the Mayo 2012 model, and the Boston University (BU) model [7,48,49]. The Mayo 2004 model and its European modification rely on TnT and NT-proBNP levels to assign risk and are most widely used to determine patient management, while the Mayo 2012 and BU models incorporate dFLC and BNP, as additional markers of disease burden, respectively [4,7,8,48]. The European modification of Mayo 2004 more accurately predicts early death than Mayo 2012; the Mayo 2012 more accurately predicts late survival than Mayo 2004 [48]. In 2024, Khwaja et al. found that incorporating myocardial global longitudinal strain (GLS) into the European modification of Mayo 2004 allowed the identification of patients at highest risk of death (“Stage IIIc”), though this model has not yet been externally verified [50]. In addition to the cardiac-based models, a renal staging model has been developed to predict the risk of progression to dialysis [8,48] (Supplementary Table 3). Additional biomarkers with predictive qualities that have not yet been incorporated into staging systems include von Willebrand factor, d-dimer, and growth differentiation factor 15 [8,48].

## Summary of the existing literature on light chain amyloidosis in Brazil

Data on AL amyloidosis diagnosis and classification in Brazil are limited, but it is clear that diagnosis is often delayed. The main causes of delayed diagnosis are that AL amyloidosis is a rare and underdiagnosed heterogeneous disease that presents with nonspecific signs and symptoms, often mimicking other diseases, as well as difficulties in detecting amyloid deposits, identifying the amyloidogenic protein, and detecting the monoclonal component. In a single center study of patients at the Hospital das Clínicas da Faculdade de Medicina da Universidade de São Paulo, Brazil, most patients (56%) were seen by ≥3 physicians before obtaining a diagnosis. Healthcare specialists visited by patients were most often general practitioners (65%), nephrologists (52%), and cardiologists (34%) [25]. The median time from symptoms onset to diagnosis was 9.0 months with 90% of patients having at least two organs involved at the time of diagnosis [25]. In 72% of cases, two or more biopsies were required to reach a diagnosis, most commonly obtained from bone marrow (57%), kidney (42%), and fat pad (38%) [25]. Techniques to identify the clonal protein were used in 80 patients (56%); within this subgroup, 66% underwent indirect immunofluorescence, 36% immunohistochemistry, and 6% mass spectrometry [25]. Standard Mayo Clinic staging was assessed in 69% of patients. Most patients (66%) were Stage III (55% were Stage IIIb) according to the European staging of advanced cardiac involvement [25]. Patients with advanced cardiac stage had the worst outcomes (median overall survival was 8.6 months for Stage III versus 52.3 months for Stage I–II – p-value  < 0.001) [25]. Most patients (84%) received chemotherapy, with a median of four cycles administered; autologous stem cell transplantation (ASCT) was performed in 14% of the cohort. Only one patient (1%) underwent kidney transplantation [25]. Of the 55 patients evaluated for hematological response, 15% achieved complete response, 9% very good partial response, 25% partial response, 25% no response, and 25% had disease progression [25].

## Questionnaire survey of light chain amyloidosis in Brazil

**Figure 2:**
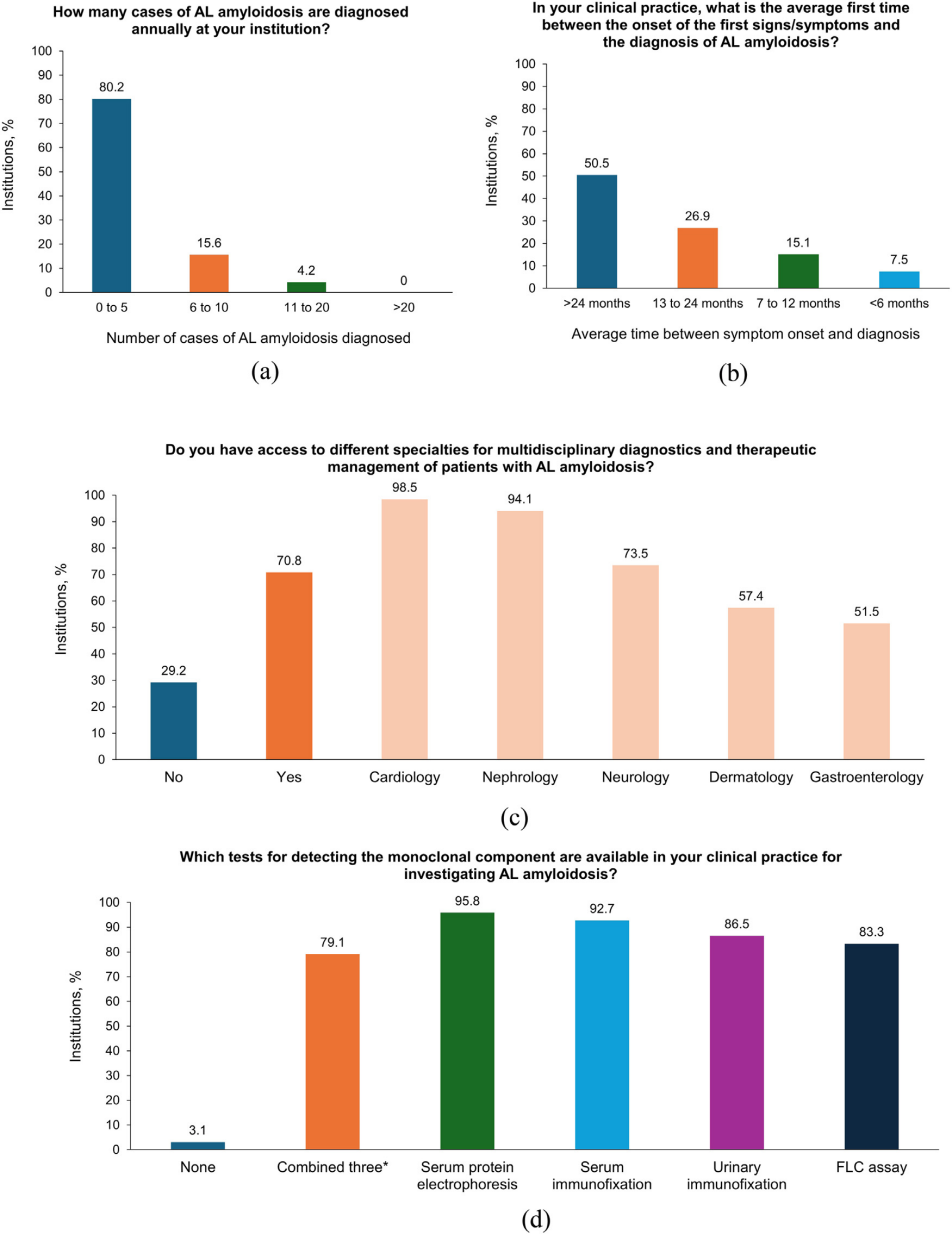

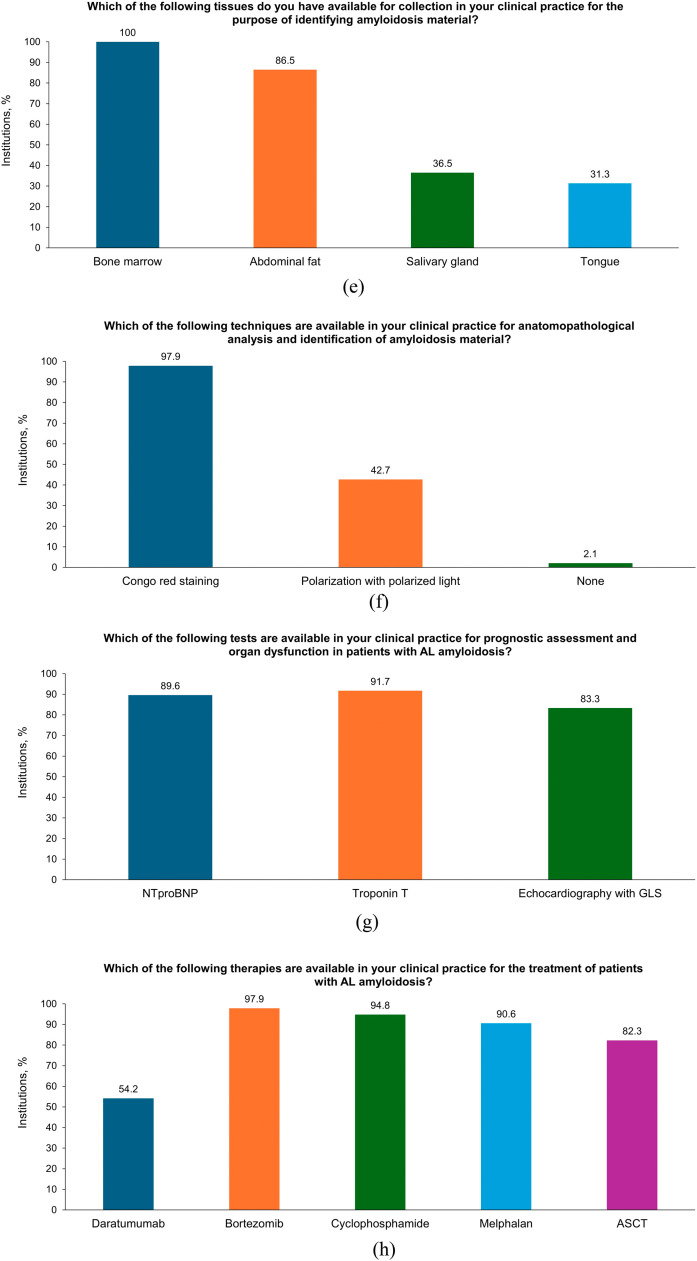
Survey results of 96 institutions across Brazil to identify the diagnostic and therapeutic challenges in AL amyloidosis *FLC assay, serum immunofixation, and urinary immunofixation. AL: Immunoglobin light chain amyloid protein; ASCT: Autologous stem cell transplant; FLC: Free light chain; GLS: Global longitudinal strain; NT-proBNP: N-terminal pro-B-type natriuretic peptide.

Responses were received from 96 institutions across the different regions of Brazil (North, Northeast, Central-West, Southeast, and South). Of this total, 52.1% were from private and 47.9% were from public institutions. The results of the questionnaire are summarized in Figure 2.

The majority (80.2%) of the institutions performed 0–5 AL amyloidosis diagnoses per year with no institution performing > 20. Regarding the interval between symptom onset and AL amyloidosis diagnosis, 7.5% of the institutions reported a timeframe of < 6 months, while 15.1%, 26.9%, and 50.5% reported delays of 7–12 months, 13–24 months, and > 24 months, respectively. Access to multidisciplinary specialties were reported by 70.7% of the institutions; the main specialties being cardiology (98.5%), nephrology (94.1%), neurology (73.5%), dermatology (57.4%), and gastroenterology (51.5%).

Most institutions (79.1%) had combined access to FLC measurement, serum immunofixation, and urinary immunofixation to detect the monoclonal component; however, access varied significantly between public (58%) and private (98%) institutions. Furthermore, 95.8% of institutions had access to serum protein electrophoresis, 92.7% to serum immunofixation, 86.5% to urinary immunofixation, and 83.3% to FLC assays; 3.1% did not have access to any of these diagnostic tools. In terms of anatomopathological analysis and identification of amyloid material, 97.9% of the institutions were capable of performing Congo red staining, 42.7% utilized polarized light microscopy to identify an apple-green birefringence; however, 2.1% did not have access to either technique. Regarding the tissue types available for amyloid detection, bone marrow biopsies were available at 100% of institutions, followed by abdominal fat pad (86.5%), salivary gland (36.5%), and tongue (31.3%) biopsies. Regarding amyloid subtyping, 76.0% of institutions had access to immunohistochemistry, 53.1% to mass spectrometry, 35.5% to immunofluorescence, and 15.6% to electron microscopy. Notably, 9.4% of institutions lacked access to any of these identification techniques.

In terms of prognostic biomarker assessment, access rates were 89.6% for NT-proBNP, 91.7% for troponin T (TnT), and 83.3% for echocardiography with GLS analysis. Combined access to all three assessments was available in 71.9% of the institutions, though a significant disparity existed between private (92%) and public (50%) centers. Notably, while NT-proBNP was available in all private institutions (100%), access dropped to 78% in the public sector.

Regarding therapeutic access for AL amyloidosis, bortezomib (97.9%), cyclophosphamide (94.8%), and melphalan (90.6%) were widely available. However, access to daratumumab was limited to 54.2% of the institutions. Furthermore, only 82.3% of the centers had the infrastructure to perform ASCT.

## Recommendations

It is clear that the management of AL amyloidosis faces a number of challenges in Brazil, including the evident disparity between public and private institutions, the need to create collaborative networks for data collection and multidisciplinary action, the long delay between clinical suspicion and diagnostic confirmation, the unavailability and low accuracy of diagnostic and prognostic tests, and limited access to therapies. Based on the available clinical evidence as well as clinical experience of the experts, a diagnostic algorithm for AL amyloidosis in Brazil is therefore proposed in Figure 1. Best-practice recommendations for the evaluation and classification of AL amyloidosis in Brazil and unanswered questions are summarized in Tables 2 and 3.

**Table 2:** Overview of expert consensus and best-practice recommendations on the diagnosis, evaluation and classification, and treatment of AL amyloidosis in Brazil.

Theme	Recommendation/consensus
Early detection	Early diagnosis of AL amyloidosis is critical to improve outcomes
	Educational initiatives need to be improved among patients, primary care physicians, and other disciplines (such as dentists and head and neck surgeons) to improve the time to diagnosis and subsequent prognosis
	The establishment of multidisciplinary specialized amyloidosis centers would allow more collaborative working to improve diagnosis
Diagnostic testing	The availability and use of diagnostic tests is variable in Brazil. This should be standardized where possible
	Procedures for what tissues to biopsy and at which timepoints need to be clear
	Transportation protocols for laboratory analysis of tissue samples should be improved and aligned to protect samples
	Training of pathology centers and the standardization of techniques in how to perform Congo red and polarized light testing consistently are required
	Involvement of the multidisciplinary approach to support biopsies is recommended. Cardiac evaluation is important to assess disease stage
	Rapid and structured diagnostic approach should follow the diagnostic algorithm in Figure 1
Evaluation and classification	Cardiac biomarker testing should be used at diagnosis, evaluation, patient follow-up, and in assessing suitability for transplant
	The tests performed at diagnosis should be used at every follow-up stage
	It is recommended to follow validated models of biomarkers and cut-offs, as well as scoring systems
	It is important to distinguish between involved and non-involved free light chains
Optimal management	A multidisciplinary approach is crucial due to the many challenges that AL amyloidosis patients experience
	The establishment of specialized amyloidosis centers should include a multidisciplinary clinical team with experience across different amyloidosis subtypes and access to disease-specific diagnostic tools and therapies
	Specialized amyloidosis centers should also include adequate infrastructure to deliver patient support services, research and educational activities, telemedicine, and collaborative networks
	The recommended treatment is the d-VCD combination, if available. or VCD where this combination is unavailable. Other options are available in low- and middle-income countries.
	The decision to use transplant should be approached with caution and with consideration of the patient’s condition. There should be alignment in the eligibility criteria for transplantation (EHA-ISA guidelines). Access to transplantation may be lower in the real-world, and eligibility is dynamic
Treatment landscape	The incorporation of new therapeutic mechanisms, such as immunotherapies (anti-BCMAs, BsAbs, and CAR-T therapy), will potentially improve therapeutic options for patients

AL: immunoglobin light chain amyloid protein; BCMA: B-cell maturation antigen; BsAbs: bispecific antibodies; CAR-T: chimeric antigen receptor T-cell; d-VCD: daratumumab with bortezomib, cyclophosphamide, and dexamethasone; EHA-ISA: European Haematology Association – International Society of Amyloidosis; VCD, bortezomib, cyclophosphamide, and dexamethasone.

**Table 3:** Unanswered questions/challenges and potential solutions/future research for the management of AL amyloidosis in Brazil.

Theme	Unanswered questions and challenges
Diagnosis	Epidemiologic data on AL amyloidosis are limited due to the absence of comprehensive population databases
	Adequate monitoring of light chain MGUS using biomarker tests (e.g., alkaline phosphatase, proteinuria, and NT-proBNP) is required for the early detection of AL amyloidosis – to allow prompt recognition of ‘red-flags’, such as fatigue, weight loss, cardiomyopathy, proteinuria, and peripheral neuropathy
	Delayed diagnosis is often due to low clinical suspicion; additional education is required
	Diagnostic resources for amyloid detection and protein characterization are scarce in many regions (e.g., abdominal fat biopsy and endomyocardial biopsy)
	Sensitivity of peripheral tissues in detecting amyloid deposits can be low in clinical practice (e.g., abdominal fat pad biopsy)
	There is inconsistency in the laboratory screening process across centers and regions
	There is limited access across different specialties
Evaluation and classification	There is inconsistency among centers and regions on what tests are being performed and at which timepoints
	There is limited access to of cardiac necrosis biomarker testing – different troponin subtypes (new generations)
	Limited access to and standardization of ECO with global longitudinal strain
Treatment and response assessment	Limited access to the best first-line treatments such as stem cell transplantation and daratumumab
	Lack of effective therapeutic options in the context of relapsed and refractory AL amyloidosis
	There is a need for a multidisciplinary approach for adequate supportive care
	Further clinical trials are needed to support future therapies in Brazil

AL: immunoglobin light chain amyloid protein; ECO: echocardiography; MGUS: monoclonal gammopathy of undetermined significance; NT-proBNP: N-terminal pro-B-type natriuretic peptide.

### Early detection

Patients with AL amyloidosis in Brazil face significant diagnostic delays, often consulting multiple HCPs including general practitioners, nephrologists, and cardiologists and at the time of diagnosis they typically present with multi-organ involvement. Early diagnosis of AL amyloidosis is critical to improve outcomes and prevent organ damage. Educational initiatives for patients and HCPs and the establishment of multidisciplinary specialized amyloidosis centers are needed in Brazil to improve the time to diagnosis and subsequent prognosis of patients. In line with recent International Society of Amyloidosis (ISA) recommendations, such centers should be structured to include a multidisciplinary clinical team; adequate infrastructure and access to diagnostic tools; expertise across different amyloidosis subtypes; access to disease-specific therapies; patient support services; research and educational activities; telemedicine capabilities; and integration within collaborative networks [51].

### Diagnostic testing

Patients in Brazil often require ≥2 biopsies for the diagnosis of amyloidosis. Improving the timeliness and accuracy of amyloidosis diagnosis requires expanded availability of monoclonal protein screening, prompt access to tissue biopsy, and standardized recommendations for biopsy site selection and timing. Additional training of pathology centers in how to consistently perform Congo red and polarized light testing is required. The use of a multidisciplinary team approach to support biopsies is recommended. Furthermore, standardization is needed for transportation protocols, for laboratory analysis of tissue samples, and for the availability and use of diagnostic tests.

### Evaluation and classification

Cardiac involvement in AL amyloidosis is associated with the worst outcome. Cardiac biomarker testing should be used at every stage, including diagnosis, prognostic evaluation, patient follow-up, and assessing suitability for transplantation. Validated models of biomarkers and cut-offs, as well as scoring systems, should be followed to standardize evaluations. Identification and quantification of involved FLC is important for biomarker testing and prognostic classification and response assessment.

### Optimal management and treatment

A multidisciplinary approach is crucial to support AL amyloidosis patients through the many challenges they experience. The main goal of treatment is to achieve a rapid and deep hematological remission by eliminating or reducing the amyloid-producing clonal cell population [4,52]. A degree of recovery in organ function occurs after circulating FLCs have been significantly reduced; recovery can continue to improve for up to two years after treatment, even when the amount of amyloid deposit remains unchanged [53, 54, 55, 56]. Given this, if an adequate hematological response (very good partial response or higher) is achieved, it is recommended to avoid premature therapeutic changes despite a lack of organ response within the first few months. Organ non-response is more common with more advanced disease and when more than one organ is involved [53,54].

**Figure 3:**
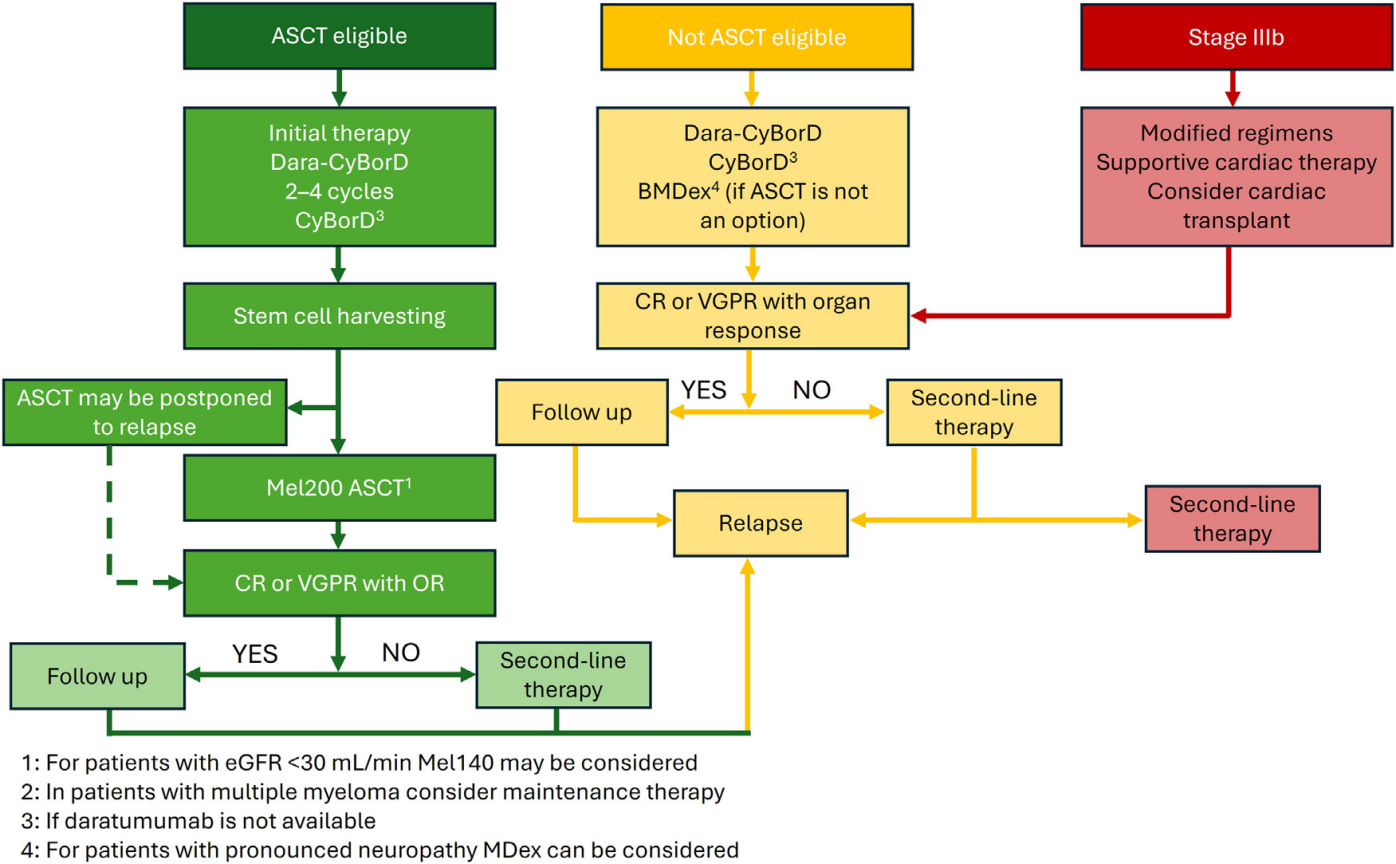
Therapeutic algorithm for the treatment of AL amyloidosis* *In cases where daratumumab is unavailable, the bortezomib-cyclophosphamide-dexamethasone should be chosen. ASCT: Autologous stem cell transplant; BMDex: Bortezomib, melphalan, and dexamethasone; CR: Complete response; CyBorD: Cyclophosphamide, bortezomib, dexamethasone; Dara: Daratumumab; eGFR: Estimated glomerular filtration rate; MDex: Melphalan and dexamethasone; Mel140: melphalan 140 mg/m²; OR: Organ response; VGPR: Very good partial response.

Management of AL amyloidosis involves supportive care and plasma cell-targeting therapies derived from multiple myeloma protocols, utilizing a similar risk-adapted approach [52]. The current standard of care for newly diagnosed AL amyloidosis is the quadruplet regimen consisting of daratumumab, bortezomib, cyclophosphamide, and dexamethasone. Approved by the Brazilian Health Regulatory Agency (ANVISA), this is also the only treatment approved by the Food and Drugs Administration (FDA) in the United States [57,58]. Standard treatment for AL amyloidosis from the late 1970s through the early 2000s involved the alkylating agent melphalan initially in combination with the corticosteroid prednisone and later with dexamethasone [59]. While melphalan-dexamethasone continues to be an effective treatment [60], hematological response rates and overall survival improve when bortezomib is added [46,61]. Results of the ANDROMEDA study show that adding daratumumab to the combination of bortezomib-cyclophosphamide-dexamethasone regimen further improved the speed and depth of hematological response, resulting in a clinically meaningful and significant improvement in overall survival and survival free from major organ deterioration [62,63]. An upfront treatment algorithm for AL amyloidosis is shown in Figure 3 [64,65]. In cases where daratumumab is unavailable, the bortezomib-cyclophosphamide-dexamethasone should be used. As mentioned, in Brazil, daratumumab is approved for the treatment of AL amyloidosis, but access is only available to patients with supplementary health assistance [66]. Most patients from public institutions receive bortezomib, cyclophosphamide, melphalan and dexamethasone; the addition of daratumumab, when available, is recommended.

Due to the high risk of mortality, ASCT is generally restricted to younger individuals with limited cardiac involvement, although evidence is lacking for its superiority over chemotherapy alone [57,67]. Indeed, only a small proportion of patients (around 20%) with AL amyloidosis are eligible for ASCT [4]. Furthermore, major shifts in ASCT utilization have occurred since the introduction of daratumumab, with some centers experiencing an 80% decline in treatment volume [68]. The decision to use ASCT should be approached with caution and should take into account the patient’s condition and the experience of the centers. Patient selection for ASCT should ideally follow risk-adapted recommendations, such as the European Hematology Association–International Society of Amyloidosis (EHA-ISA) guidelines for stem cell transplantation in systemic AL amyloidosis [69]. ASCT should not be offered to patients with: advanced cardiac involvement (particularly Stage IIIb disease), medically refractory pleural effusions, refractory orthostatic hypotension, active gastrointestinal bleeding, or acquired factor X deficiency with active bleeding. Conditioning melphalan should be dose-adjusted (200 mg/m² or 140 mg/m²) according to age, cardiac status, renal function, and overall condition. Similarly stringent eligibility criteria have been published by the Mayo Clinic [32]. Solid organ transplantation may be an option for patients with more advanced disease, particularly with renal involvement, but again this is not well supported by available data [70,71]. Use of immunomodulatory drugs, such as lenalidomide, or proteasome inhibitors (e.g., carfilzomib, ixazomib) alone or in combination is limited, due to the increased potential for cardiotoxicity and lack of evidence of efficacy [26]. Agents that directly target fibril formation and amyloid deposits at early stages of clinical development [72] are not currently available for clinical use. Although Phase 3 studies have been conducted for anti-amyloid agents such as birtamimab and anselamimab, these trials did not meet their primary endpoints [73,74].

Regarding second-line treatment, daratumumab rechallenge can be considered, with bortezomib re-exposure also being an option in certain circumstances. Patients who achieve a durable, well-tolerated response to a bortezomib-containing regimen, particularly in the absence of neuropathy, may be considered for proteasome inhibitor re-exposure, ideally employing a different combination. For patients who have not previously received anti-CD38 therapy, daratumumab-based regimens represent a reasonable option [75]. Also, enrollment in clinical trials should be encouraged if feasible [76,77]. While not currently FDA approved, venetoclax is an investigational second-line treatment option for patients with AL amyloidosis with the t(11;14) translocation [78]. Venetoclax has demonstrated good efficacy in patients with relapsed or refractory AL amyloidosis harboring the t(11;14), although its availability remains largely restricted to clinical trials [78,79]. In a multicenter, retrospective analysis of 22 patients with AL amyloidosis treated with venetoclax, the median number of prior lines of therapy was one (range: 1–3). The most common frontline regimen was daratumumab, bortezomib, cyclophosphamide, and dexamethasone (68%), with 22% of patients having previously undergone ASCT. All 22 patients achieved a complete response or very good partial response with venetoclax, though nine patients eventually progressed before or after venetoclax discontinuation [80].

### Assessing response to therapy

Response to therapy is assessed in terms of hematological response and organ-specific responses, with cardiac involvement being the most important factor in early mortality [46]. Hematological response is measured as the difference between involved and uninvolved free light chains (dFLC) [26,46] and organ response is assessed by measuring organ-specific biomarkers [26,46]. Supplementary Table 4 summarizes the criteria for evaluating hematologic response and organ response to therapy [48]. A significant decrease in dFLC is associated with improved clinical outcomes; specifically, achieving a dFLC < 10 mg/L predicts superior overall survival, higher organ response rates, and a longer time to next treatment [26,46]. Assessment of hematological response should be conducted at least every three months while on active treatment and after completion of treatment; proper monitoring should begin early, especially in the initial treatment phase [26]. As rising dFLC levels per se do not uniformly mandate the initiation of second-line therapy, an individualized approach is needed; some patients may experience prolonged periods without organ progression after hematologic relapse. In a Mayo Clinic study, 23% of patients had no evidence of organ disease progression for a median of 14 months and up to 8.3 years [81]. However, the optimal timing of treatment at relapse remains an area of active debate. Some experts advocate earlier intervention to prevent organ damage [82]. Accordingly, more recent consensus recommendations suggest that, in patients who previously achieved a very good partial response defined as the dFLC < 40 mg/L or better, salvage therapy should be considered in the presence of rising dFLC levels before overt organ progression develops, even if hematologic progression is not seen [32].

In cases of relapsed and refractory AL amyloidosis, treatment options are highly individualized with no universally accepted treatment sequence. While some patients experience slow biochemical relapse without immediate organ deterioration, management must be tailored to prior organ involvement, patient frailty, and the kinetics of light-chain progression. This is best captured by the concept of **‘**high-risk dFLC progression’ (dFLC > 20 mg/L, representing > 20% of the baseline and a > 50% increase from the nadir) [83]. Such a personalized strategy is preferred over a uniform ‘watch and wait’ approach. Guidelines developed by the EHA-ISA working group emphasize balancing early treatment to prevent serious organ damage, particularly in patients with significant cardiac, renal, or autonomic involvement, against the risks of premature re-initiation of chemotherapy in frail patients, underscoring the need for individualized clinical judgment [84]. While the optimal sequencing of therapies in relapsed or refractory AL amyloidosis cannot be prescriptive, treatment decisions should be guided by the depth and duration of the initial hematologic response, prior exposure to specific drug classes, and the constraints related to the patient’s condition and the extent of end-organ involvement.

## Discussion and future direction

Data on AL amyloidosis are generally scarce in middle- and low-income countries, and there are currently no Brazilian guidelines for the treatment of AL amyloidosis. In contrast, real-world evidence exists across Europe [85] and several region-specific consensus recommendations or guidelines exist to optimize the management of AL amyloidosis [4,26, 27, 28, 29, 30,32,84]. Here, we summarize the available evidence for AL amyloidosis in Brazil from the literature, report contemporary data from a recent survey of Brazilian institutions, and provide expert-guided practical guidance and recommendations for the optimal diagnosis, evaluation and classification, and treatment of AL amyloidosis in Brazil.

The results from the recent survey provide contemporary insights and highlight gaps in the diagnosis and management of AL amyloidosis in Brazil. First, the low diagnosis rates likely reflect the epidemiological rarity of AL amyloidosis as well as challenges in its recognition, suggesting that the disease is underdiagnosed in Brazil. In Brazil, the low identification rates and diagnostic delays in AL amyloidosis are likely driven by clinical heterogeneity alongside systemic barriers, such as the complexity of the workup and limited access to specialized tools. Given the clinical heterogeneity of AL amyloidosis, a multidisciplinary approach is critical for accurate diagnosis and to reduce the delay; however, 29.2% of institutions in Brazil did not have access to different specialties in diagnostic and therapeutic management. The survey also highlights the gaps in the fundamental diagnostic testing of AL amyloidosis in Brazil: 20.9% of institutions did not have access to all three tests (FLC assay, serum immunofixation, and urinary immunofixation) to detect the monoclonal component and 3.1% did not have access to any of these tests. Moreover, 2.1% did not have access to any of the anatomopathological techniques for identifying amyloid material, and 9.4% did not have access to amyloid protein typing techniques. These missing diagnostic techniques pose a significant limitation to the accurate and timely diagnosis and staging of AL amyloidosis in Brazil. Because the prognosis of AL amyloidosis is directly linked to the timing of interventions, a delayed diagnosis often results in advanced cardiac involvement and, consequently, inferior survival outcomes [86]. Finally, the survey highlighted that there is still considerable heterogeneity in access to new AL amyloidosis therapies across Brazilian institutions, chiefly for those within the public health system. In particular, only 54.2% of institutions had access to daratumumab for AL amyloidosis, despite daratumumab plus bortezomib-cyclophosphamide-dexamethasone being the standard of care endorsed by the ANDROMEDA study [62,63].

The expert recommendations provided here aim to educate and aid specialist and community physicians in Brazil who are involved in the treatment of patients with AL amyloidosis, as well as the wider clinical teams who may support the early identification and diagnosis of patients (such as cardiologists and nephrologists). Despite the practical guidance provided here, there remain unanswered questions and challenges that impede the optimal diagnosis and management of AL amyloidosis in Brazil (Supplementary Table 4).

A key strength of this survey is the inclusion of 96 institutions spanning all Brazilian regions and both public and private healthcare sectors, providing a comprehensive view of the diverse challenges in managing AL amyloidosis. Furthermore, these consensus recommendations consolidate the collective expertise of a multidisciplinary group of specialists from across the country. Elements of the NGT were implemented to capture expert consensus on AL amyloidosis in a structured, balanced, and clear manner. A limitation of the survey and the consensus recommendations is that they are representative of the management of AL amyloidosis in Brazil and therefore may not be applicable to countries with different treatment options or healthcare systems.

### Diagnosis and treatment of localized light chain amyloidosis

Localized AL amyloidosis accounts for around 10% of all amyloidosis cases [87]. In the clinical practice, localized AL amyloidosis may be misdiagnosed as systemic AL amyloidosis, leading to chemotherapy treatment that is not clinically indicated. Correct staging and diagnosis are therefore essential to avoid intensive treatment for what is often a curable condition. Localized AL amyloidosis presentation is variable, depending on the specific organ involved [88]. Diagnosis of localized AL amyloidosis differs from the systemic form in that it only requires detection of amyloid deposits and identification of the amyloid protein. An initial hematologic evaluation for a monoclonal protein is typically sufficient to rule out systemic AL amyloidosis [88]. In localized AL amyloidosis, there is typically no evidence of a circulating monoclonal protein or systemic organ involvement. Consequently, these patients must be accurately distinguished from those with systemic disease to ensure they receive an appropriate site-specific therapy.

Management of localized disease can be challenging [89]. The goal of treatment for patients is symptom control [88]. Surgical resection is the most common first-line treatment [88,89], while radiotherapy can stabilize refractory and locally advanced disease that is not suitable for surgery. In some cases, a watch and wait strategy may be appropriate. Localized AL amyloidosis usually does not progress to systemic AL amyloidosis [90], which should directly impact how clinical and laboratory follow-up is performed in practice [91].

## Conclusions

Developed through structured virtual advisory exchanges, this article provides expert consensus and practical recommendations for the optimal diagnosis, evaluation, and treatment of AL amyloidosis in Brazil. These guidelines serve as a primary reference for healthcare professionals (HCPs) and outline strategic solutions and research priorities aimed at strengthening the evidence base and improving patient outcomes in Brazil.

## Author contributions

All authors were responsible for idea generation, critical review, and input into the manuscript drafts, as well as approval of the final draft for submission.

## Data availability

All data are available within the text.

## Conflicts of interest

Pedro Manoel Marques Garibaldi: Honoraria from GSK, Johnson & Johnson, Pfizer

João Tadeu Damian Souto Filho: Honoraria from AbbVie, AstraZeneca, BeOne, Johnson & Johnson, Sanofi

Roberto Jose Pessoa de Magalhães Filho: Honoraria from Johnson & Johnson

Roberta Shcolnik Szor: Honoraria from Johnson & Johnson

Carlos Miguel: Honoraria from Johnson & Johnson

Edvan Crusoe: Honoraria from Amgen, Bristol Myers Squibb, GSK, Johnson & Johnson, Natcofarma, Sanofi, Takeda

Carolina Tosin Bueno and Thiago Queiroz: Johnson & Johnson employees

Vânia Hungria: Honoraria from Amgen, AbbVie, BMS, GSK, Johnson & Johnson Pfizer, Regeneron, Roche, Sanofi, Takeda

## Appendix Supplementary materials

Image, application 1

Image, image 2 dummy alt text
