# Supplementary material for: Brazilian consensus recommendations on the diagnosis and treatment of light chain amyloidosis
Source: Hematol Transfus Cell Ther. 2026 Jun 2;48(3):106482. doi: 10.1016/j.htct.2026.106482 (PMC13251756; doi:10.1016/j.htct.2026.106482)
Supplement: Supplementary file 1 [file mmc1.docx]

# Supplementary Table 1: Different forms of systemic amyloidosis with major organ involvement

| **Amyloid type** | **Precursor protein** | **Organ involvement (Frequency)** | | | | | | |
| --- | --- | --- | --- | --- | --- | --- | --- | --- |
|  |  | **Heart*** | **Kidney** | **Liver** | **Peripheral nervous system** | **Autonomic nervous system** | **Soft tissue** |  |
| AL (acquired) | Immunoglobulin light chain | ≥50% (usually absent, can be intense) | ≥50% | 10%-30% | ≤10% | ≤10% | 10%-30% |  |
| ATTRv (hereditary) | Mutated transthyretin | ≥50%  (usually intense, can be absent in some variants) | Rare or not involved | Rare or not involved | ≥50% | ≥50% | Rare or not involved |  |
| ATTRwt (acquired) | Wild-type transthyretin | ≥50% (usually intense) | Rare or not involved | Rare or not involved | Rare or not involved | Rare or not involved | ≤10% |  |
| ApoAI (hereditary) | Mutated apolipoprotein A1 | ≤10% (present) | ≤10% | ≥50% | Rare or not involved | Rare or not involved | Rare or not involved |  |
| AA (acquired) | Serum amyloid A protein | ≤10% | ≥50% | ≤10% | Rare or not involved | ≤10% | Rare or not involved |  |
| ALECT2 (acquired) | Leukocyte chemotactic factor 2 | Rare or not involved | ≥50% | ≤10% | Rare or not involved | Rare or not involved | Rare or not involved |  |

Table adapted from [Palladini 2020].^4^
*bone tracer uptake.
AA: amyloid A; AL, immunoglobin light chain amyloidosis: ALECT2, amyloidosis derived from leukocyte chemotactic factor 2; ApoAI: apolipoprotein A1; ATTRv: hereditary transthyretin amyloidosis; ATTRwt: wild-type transthyretin amyloidosis.

**Supplementary Table 2: Summary table of AL amyloidosis**

| **Organ** | **Frequency of involvement** | **Consensus criteria for involvement^a^** | **Common presenting signs and symptoms** | **Diagnostic findings** |
| --- | --- | --- | --- | --- |
| Heart | 60–75% | NT-proBNP >332 ng/L^c^ OR mean IVSd >12 mm | - Angina^b^ - Arrhythmia - Dyspnea or exertion - Jugular venous distention - Lower extremity edema - Orthopnea - Paroxysmal nocturnal dyspnea - Pleural effusions - Syncope | ECG   - Low QRS voltage - Conduction system disease - Atrial fibrillation - Poor R-wave progression in precordial leads   TTE   - Increased wall thickness - Diastolic dysfunction with preserved LVEF - Reduced GLS - CMR - Late gadolinium enhancement - RHC - Restrictive physiology |
| Kidney | 50–70% | Proteinuria ≥0.5 g/24 hours (mostly glomerular proteinuria, this albumin) | - Anasarca - Lower extremity edema - Uremia | - Globular proteinuria (albuminuria) - Acute kidney injury - Hypercholesterolemia - Hypercoagulability |
| Lung^f^ | 30–90%^f^ | Direct biopsy verification | - Dry cough - Recurrent pleural effusions - Shortness of breath | - Pleural effusions - Interstitial pulmonary nodules |
| Liver | 20% | Liver span >15 cm^e^ OR alkaline phosphatase elevation <1.5 times ULN | - Early satiety - Right upper quadrant tenderness - Weight loss | - Hepatomegaly - Isolated increase in alkaline phosphatase - Coagulopathy caused by coagulation factor deficiency^d^ |
| Gastro-intestinal tract | 10–20% | Direct biopsy verification | - Diarrhea - Hematochezia or melena - Malabsorption - Weight loss |  |
| PNS | 10–20% | Clinical diagnosis | - Distal sensorimotor PN | - EMG: symmetric, axonal sensorimotor polyneuropathy |
| ANS | 10–20% | Clinical diagnosis | - Early satiety - Erectile dysfunction - High (pseudo-obstruction, vomiting), or low (constipation alternating with diarrhea) intestinal dysmotility - Orthostatic hypotension - Voiding dysfunction | - Delayed gastric emptying - Positive tilt test |
| Soft tissue | 10–20% | Clinical diagnosis | - Arthropathy - Carpel tunnel (often bilateral) - Ecchymotic bullae - Jaw or buttock claudication^g^ - Macroglossia - Myopathy - Periorbital (or upper body) purpura |  |

Table adapted from [Bianchi 2021].^46^
The table outlines the incidence of organ involvement and frequent signs/symptoms and diagnostic findings in patients with AL amyloidosis based on pattern of organ involvement. Consensus criteria for diagnosis is also reported. ^a^Alternative etiologies must be excluded. ^b^Typical of patients with amyloid deposition in the smaller vessels within the heart wall, mimicking coronary artery disease in the absence of large-vessel disease. ^c^In the absence of renal failure or atrial fibrillation. ^d^Factor X deficiency can occur independently of liver involvement caused by direct absorption of factor X by amyloid fibrils. ^e^In the absence of congestive hepatopathy secondary to heart failure. ^f^Depending on single institution series, often asymptomatic and detected postmortem. ^g^Presumed related to vascular deposition of amyloid.
AL: immunoglobin light chain amyloidosis; ANS: autonomic nervous system; CMR: cardiac magnetic resonance; ECG: electrocardiogram; EMG: electromyography; GLS: global longitudinal strain; IVSd: interventricular septal wall thickness at end diastole; LVEF: left ventricular ejection fraction; NT-proBNP: N-terminal pro-B-type natriuretic peptide; OR: organ response; PN: peripheral neuropathy; PNS: peripheral nervous system; RHC: right hear catheterization; TTE: transthoracic echocardiogram; ULN: upper limit of normal.**Supplementary Table 3: Most commonly used AL amyloidosis prognostic staging systems**

| **Staging system** | **Markers and thresholds** | **Stages** | **Survival outcomes*** **(median)** |
| --- | --- | --- | --- |
| Cardiac  (NT-proBNP based)^92^ | NT-proBNP >332 ng/L  cTnT >0.035 ng/mL  (or cTnl >0.01 ng/mL) | I. No markers above cutoff | I. Not reached, 57% with 10-year survival |
|  |  | II. One marker above cutoff | II. 67 months |
|  |  | IIIa. Both markers above cutoff and NT-proBNP <8500 ng/L | IIIa. 15 months |
|  |  | IIIb. Both markers above cutoff and NT-proBNP ≥8500 ng/L | IIIb. 4 months |
| Cardiac  (BNP based)^93^ | BNP >81 ng/L  cTnl >0.1 ng/mL | I. No markers above cutoff | I. 151 months, 57% with 10-year survival |
|  |  | II. One marker above cutoff | II. 53 months |
|  |  | IIIa. Both markers above cutoff and BNP <700 ng/L | IIIa. 13 months |
|  |  | IIIb. Both markers above cutoff and BNP ≥700 ng/L | IIIb. 4 months |
| Revised Mayo Clinic^94^ | NT-proBNP >1800 ng/L  cTnT >0.025 ng/mL  dFLC >180 mg/L | I. 0 markers above cutoff | I. Not reached, 57% with 10-year survival |
|  |  | II. 1 marker above cutoff | II. 69 months |
|  |  | III. 2 markers above cutoff | III. 16 months |
|  |  | IV. 3 markers above cutoff | IV. 6 months |
| Renal^95^ | eGFR <50 mL/min per 1.73 m^2^  Proteinuria >5 g per 24 hours | I. Both eGFR above and proteinuria below cutoffs | I. 1% risk of dialysis at 2 years |
|  |  | II. Either eGFR below or proteinuria above cutoffs | II. 12% risk of dialysis at 2 years |
|  |  | III. Both eGFR below and proteinuria above cutoffs | III. 48% risk of dialysis at 2 years |

Table adapted from [Palladini 2020] and [Al Hamed 2021].^4, 7^
*Observed in 1378 patients with AL amyloidosis newly diagnosed at the Pavia Amyloidosis Research and Treatment Center from 2004 through 2028.
AL: immunoglobin light chain amyloid protein; BNP: B-type natriuretic peptide; cTnI: cardiac troponin I; cTnT: cardiac troponin T; dFLC: difference between involved (amyloidogenic) and uninvolved circulating free light chain; eGFR: estimated glomerular filtration rate; NT-proBNP: N-terminal pro-B-type natriuretic peptide.

**Supplementary Table 4: Criteria for evaluation hematologic response and organ response to therapy**

| **Response type** | **Response criteria** |
| --- | --- |
| **Hematologic** | |
| - CR | Negative serum and urine IFE and normal serum immunoglobulin kappa/lambda FLC ratio with uninvolved FLC concentration greater than involved FLC concentration with/without abnormal FLC ratio |
| - VGPR | dFLC <40 mg/L |
| - PR | dFLC decrease of >50% |
| - NR | Less than a PR |
| *For patients with baseline dFLC 20−50 mg/L* |  |
| - CR | Negative serum and urine IFE and normal serum immunoglobulin kappa/lambda FLC ratio with uninvolved FLC concentration greater than involved FLC concentration with/without abnormal FLC ratio |
| - dFLC PR | dFLC <10 mg/L |
| **Cardiac** | |
| - Cardiac CR (CarCR) | Nadir NT-proBNP ≤350pg/m or BNP≤ 80 pg/mL |
| - Cardiac VGPR | >60% reduction in NT-proBNP/BNP from baseline level not meeting CarCR |
| - Cardiac PR | 31−60% reduction in NT-proBNP/BNP from baseline level not meeting CarCR |
| - Cardiac NR | ≤30% reduction in NT-proBNP/BNP from baseline level |
| **Renal** | |
| - Renal CR (RenCR) | Nadir proteinuria ≤200 mg/24h |
| - Renal VGPR | >60% reduction in proteinuria from baseline level not meeting RenCR |
| - Renal PR | 31−60% reduction in proteinuria from baseline level not meeting RenCR |
| - Renal NR | ≤30% reduction in proteinuria from baseline level |
| **Hepatic** | |
| - Response | Decrease in serum alkaline phosphatase from baseline value >50% |
| - No response | Decrease in serum alkaline phosphatase from baseline value ≤50% |

Table adapted from [Sanchorawala et al.]^48^

BNP: B-type natriuretic peptide; CR: complete response; dFLC: difference between involved (amyloidogenic) and uninvolved circulating free light chain; FLC: free light chain; IFE: immunofixation electrophoresis; NR: no response; NT-proBNP: N-terminal pro-B-type natriuretic peptide; PR: partial response; VGPR: very good partial response.

**Supplementary Figure 1: Clinical signs, symptoms, and laboratory abnormalities of AL amyloidosis**

ANS: autonomic nervous system; GI: gastro-intestinal; HFpEF: heart failure with preserved ejection fraction; LVH: Left ventricular hypertrophy.
