# Supplementary figures and images for: Brazilian consensus recommendations on the diagnosis and treatment of light chain amyloidosis
Source: Hematol Transfus Cell Ther. 2026 Jun 2;48(3):106482. doi: 10.1016/j.htct.2026.106482 (PMC13251756; doi:10.1016/j.htct.2026.106482)

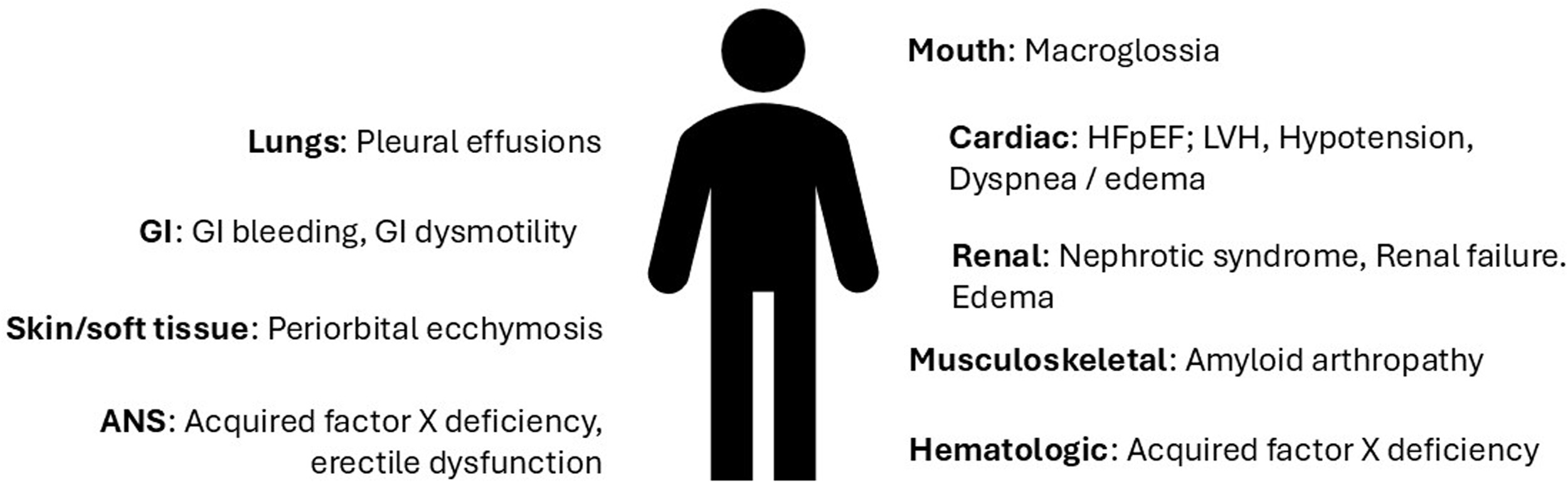

Supplement: Supplementary file 2 [file mmc2.jpg]
